# Myoinositol in the Prevention of Gestational Diabetes Mellitus: Is It Sensible?

**DOI:** 10.1155/2019/3915253

**Published:** 2019-12-07

**Authors:** Angelika Sobota-Grzeszyk, Mariusz Kuźmicki, Jacek Szamatowicz

**Affiliations:** Department of Gynecology and Gynecological Oncology, Medical University of Bialystok, 15-276 Bialystok, Poland

## Abstract

Gestational diabetes mellitus (GDM) is a complication that increasingly affects pregnant women. Due to the risk of adverse outcomes in the mother as well as in the fetus which is caused by GDM, appropriate diagnosis and treatment is very essential. Nevertheless, it is important to find new, effective ways of prevention of GDM to avoid side effects. A promising example of such an action may be supplementation of myoinositol. As shown in studies, myoinositol may reduce the risk of developing gestational diabetes mellitus by improving insulin sensitivity.

## 1. Introduction

Gestational diabetes mellitus (GDM) is a growing problem around the world. It is characterized by insulin resistance, but this mechanism is not fully understood [1]. These disorders usually disappear after delivery, but as per the data provided, in approximately 50% of patients who were diagnosed with gestational diabetes, in the next few years, these will reveal the impaired glucose tolerance or type 2 diabetes [2]. In addition, GDM is associated with maternal and perinatal morbidity including fetal macrosomia, shoulder dystocia, neonatal hypoglycemia and adiposity, respiratory distress syndrome, the need to terminate pregnancy by caesarean section, hypertensive disorders, and preeclampsia [3–5]. Moreover, GDM is connected with increased risk of developing type 2 diabetes later in life for the mother. Women who have diabetes mellitus during pregnancy have high levels of insulin resistance during the postpartum period or later in life which may indicate that GDM is a manifestation of glucose tolerance disorders with the increased risk of relapse in the future [6]. These complications can be reduced by finding new, simple, acceptable, and effective methods of prevention of gestational diabetes for pregnant women which can control the mother's blood glucose levels without hurting the mother or the fetus. One of these methods may be myoinositol supplementation.

The results of one of the studies indicate that myoinositol reduces insulin resistance; therefore, it can also be used in gestational diabetes [7]. Other researchers report that myoinositol may have a stimulating effect on endogenous insulin [8, 9]. The reports from these studies suggest that myoinositol may find an application in the prevention of gestational diabetes.

## 2. The Role and Sources of Myoinositol

Inositol is a polyol which due to epimerization can occur in nine stereoisomeric forms depending on the distribution of its six hydroxyl groups [10]. It is considered as pseudovitamin and belongs to the vitamin B complex; however, defining inositol as a vitamin is not completely correct because it is produced in sufficient amount by the human body from D-glucose. It occurs in the liver, kidneys, and brain [11, 12]. Inositol was described as a second messenger system that may exert an insulin-like effect on metabolic enzymes and consequently improve insulin sensitivity [13–15].

The most important inositol isomers are myo-, chiro- (L-chiro and D-chiro), muco-, scyllo-, and neo-. Myoinositol, also known as a 1,2,3,5-trans-4,6-cyclohexanehexol, is a predominant isomeric form of inositol that is found in animal and plant cells. It occurs in its free form or as a component of phospholipids or as phytic acid. Myoinositol is a component of the membranes of all living cells. It also plays a role in the synthesis of lipids. Myoinositol is found in natural dietary sources such as beans, nuts, and cereals. The highest concentrations of myoinositol contain fresh citrus fruits (excepted lemons), vegetables, and food including seeds.

Both myoinositol and D-chiroinositol have already found their use in the polycystic ovary syndrome and in type 2 diabetes [7, 11, 16].

## 3. Myoinositol in the Prevention of GDM: Randomized Trials

The prevention of GDM is very important because of the teratogenic influence of high-glucose concentration on the fetus. This may result in abnormalities in fetal development and have adverse effects on the offspring.

After it was reported that myoinositol improves insulin sensitivity in patients with PCOS, researchers began to analyze whether myoinositol can prevent or reduce insulin resistance in patients at risk for developing or with gestational diabetes. There are few studies on the role of myoinositol in pregnant women at risk for GDM. In four randomized studies, the researchers tested myoinositol in pregnant women at risk for gestational diabetes mellitus [8, 9, 17]. There is also one study investigating the effect of myoinositol in comparison with D-chiroinositol and manganese [18].

In the first study, performed by Corrado et al., pregnant women with GDM received myoinositol supplementation (2 g twice/d) plus folic acid (200 mcg twice/d) in a study group and folic acid only (200 mcg twice/d) in a control group with a controlled diet for 8 weeks. Researchers studied fasting glucose and insulin and consequently insulin resistance expressed as insulin resistance by the homeostasis model assessment (HOMA-IR). HOMA-IR was similarly reduced in the study group compared with the control group (*p* = 0.0001), while fasting serum insulin and blood glucose levels improved more in women receiving myoinositol. Furthermore, during the treatment with myoinositol, the researchers noticed an increase in adiponectin levels from basal (12.8+−5.1 basal vs 16.1+−6.6; *p* = 0.009), while levels in adiponectin in the control group were reduced (12.2+−4.6 basal vs 11.3+−4.8; difference not statistically significant) [8].

D'Anna et al. performed a prospective randomized open-label placebo-controlled trial in pregnant women with a family history of type 2 diabetes in first-degree relatives. The exclusion criteria were BMI > 30 kg/m^2^, history of PCOS, GDM or DM, and multiple gestation. Patients accepted a dietary supplement of myoinositol (2 g plus 200 mcg folic acid twice a day) or only 200 mcg folic acid twice a day throughout pregnancy. Women are randomized to the study or control group between 12 and 13 weeks of pregnancy. They had OGTT at 24 to 28 weeks. There were statistically significant reductions of the rate of gestational diabetes mellitus demonstrated by an oral glucose tolerance test (OGTT) (6% cases in a study group vs 15.3% cases in a control group) (*p* = 0.04). Furthermore, myoinositol was responsible for the reduction of fasting and 1-hour glycemia at OGTT (*p* = 0.001 and *p* = 0.02, respectively). Moreover, the offspring of the study group had a lower birth weight [9].

Using the same methodology, D'Anna et al. performed another randomized open-label placebo-controlled trial in obese pregnant women exposed to myoinositol supplementation. Exclusions were history of GDM or DM, multiple gestation, prepregnancy hypertensive, corticosteroid therapy, or renal diseases. Patients from 2 Italian hospitals were allocated to the study or control group. They received 2 g myoinositol +200 mcg folic acid twice a day (study group) or 200 mcg folic acid twice a day (control group) throughout pregnancy. The rate of GDM in the study group was significantly lower than that in the placebo group (14.0% vs. 33.6%, respectively; *p* = 0.001). Furthermore, reduction in HOMA-IR in the group of women treated with myoinositol also showed decreased insulin resistance (−1.0 ± 3.1 vs. 0.1 ± 1.8; *p* = 0.048). Moreover, there were statistically significant reductions of the gestational hypertension and the need for treatment in the neonatal intensive care unit in the study group [19].

Matarrelli et al. carried out the prospective randomized placebo-controlled double-blind study which also showed the relationship between myoinositol supplementary and gestational diabetes mellitus. They included nonobese women with elevated fasting glucose levels (92-126 mg/dL) early in pregnancy. Patients were randomized to receive 2 g myoinositol plus 200 mcg folic acid twice a day in a study group and 200 mcg of a folic acid twice a day in a control group. 6% of the study group had abnormal maternal glucose levels by OGTT compared with 71% of the control group. 1 case in the group with myoinositol and 8 cases in the group with placebo needed insulin therapy (*p* = 0.053). Researchers found that the supply of myoinositol was associated with lower risk of hyperglycemia complications: increased amniotic fluid, increased fetal abdominal circumference, macrosomia, and early delivery. Unfortunately, these difference were not statistically significant [17].

Malvasi et al. performed a prospective randomized double-blind placebo-controlled study in which healthy pregnant women were randomized to receive 2 g myoinositol plus 400 mcg D-chiroinositol plus 400 mcg folic acid plus 10 mg manganese daily or placebo for 20 days. After 30 and 60 days, researchers examined levels of maternal blood pressure, BMI, serum glucose, total cholesterol, HDL-cholesterol, LDL-cholesterol, and triglycerides. After 30 days, the level of serum glucose was reduced from 81+−5.6 to 77.3+−4.1 mg/dL and continued to be stable at 77.4+−4.2 mg/dL after 60 days of treatment (*p* = 0.019). There was statistically significant reduction compared with the placebo group where the values of mean fasting glucose increased from 79.7+−7.7 to 82.2+−6.2 mg/dL after 30 days and 82.3+−7.2 mg/dL after 60 days (*p* = 0.002 at 30 days and *p* = 0.006 at 60 days; myoinositol vs. placebo). Furthermore, after 30 days, total cholesterol (*p* = 0.0001), LDL-cholesterol (*p* = 0.0013), triglycerides (*p* < 0.0001), and glycemia (*p* = 0.0021) were statistically significantly reduced. After 60 days, a significant difference was observed for glycemia (*p* = 0.0064), total cholesterol (*p* = 0.0001), LDL (*p* = 0.0001), HDL (*p* = 0.0001), and TG (*p* = 0.0001). Moreover, after 30 as well as 60 minutes, there were no significant differences for systolic and diastolic blood pressure [18].

Santamaria et al. conducted an open-label controlled trial that randomized overweight women to myoinositol vs. placebo. The exclusion criteria were history of DM, first trimester glucosuria, corticosteroid therapy, and multiple gestation. The incidence of GDM was significantly reduced in the myoinositol treatment group compared with placebo (11.6% vs. 27.4%; *p* = 0.004). The secondary outcomes were cesarean section rate, birthweight > 4000 g, neonatal intensive care unit admission, and other, but there was no statistically significant difference [20] (Table 1).

Cochrane collaboration carried out a meta-analysis regarding supplementation with myoinositol in women during pregnancy for preventing GDM. Authors included four randomized controlled studies yielding 567 women fulfilling the criteria: two open-label trials by D'Anna et al. described above; a study by Malvasi et al. using myoinositol with D-chiroinositol, folic acid, and manganese; and a trial that reported only interim analysis. Three of these studies may be used for the analysis of the effect of myoinositol on gestational diabetes mellitus. Malvasi et al. investigated an application of myoinositol in euglycemic women; therefore, this study cannot be subjected to such analysis. Based on this analysis, it can be stated that the incidence of GDM ranged from 8% to 18% in the group of myoinositol supplementation, while in the placebo group, it was 28%. There was no significant difference in the risk of hypertensive disorders of pregnancy, fetal macrosomia, neonatal hypoglycemia, shoulder dystocia, or cesarean section rates between the myoinositol and control groups. The authors of this meta-analysis pointed to the weaknesses of these studies. All four trials were performed in Italy and done only on Caucasian women and thus did not take into account ethnic differences. Moreover, 3 of the 4 trials included in the meta-analysis are open-label. The evidence from these studies is based on small trials and is not strong enough to detect significant differences in neonatal and childhood outcomes. Furthermore, the women in the intervention groups used diet and folic acid besides myoinositol which would have confused the profitable effects of myoinositol. Nevertheless, the authors concluded that the myoinositol supplementation during pregnancy is beneficial for reducing the risk of GDM [21].

In the above trials, the study group took 4 g of inositol daily. There is also a study that applies the combination of 1,100 mg of myoinositol and 27.6 g of D-chiroinositol daily compared to 4 g of myoinositol daily in pregnancy to prevent development of gestational diabetes [22]. Researchers assumed that this combination of myoinositol and D-chiroinositol reflects the physiological ratio of inositol in the human body. It was found that this combination dose may be insufficient to prevent GDM. The higher doses of myoinositol seem to be safe and effective. The authors note that myoinositol supplementation alone may have a better effect on the glucose metabolism compared to the combination of myoinositol and D-chiroinositol. It is certainly necessary to test varying doses of myoinositol to assess their clinical efficacy during pregnancy [23].

## 4. Conclusions

The prevention of GDM is very important because high-glucose levels may lead to disturbances in construction, function, and development of the fetus. Therefore, maintaining normal levels of glucose during pregnancy is very important. The above studies present positive effects of fighting insulin resistance from the use of myoinositol.

Myoinositol is involved in many biological processes such as growth and survival of cells, development and function of peripheral nervous, osteogenesis, reduction of LDL-cholesterol and triglycerides, and increase of HDL-cholesterol. It also plays an important role in reproduction by improving oocyte quality, restoring ovulation and fertility, decreasing hyperandrogenism, and insulin resistance. Due to these properties, myoinositol is used in women with polycystic ovary syndrome [7, 10, 11]. While the molecular mechanism of myoinositol actions is not completely understood, it is thought that myoinositol binds to glucosamine to create inositol phosphoglycan-adenosine monophosphate kinase inhibitor (IPG-A) which leads to an increase in fatty acid synthesis by inhibiting c-AMP-dependent protein kinase and adenylate cyclase. Moreover, it is believed that myoinositol due to its insulin-mimetic qualities activates GLUT-4 translocation to the plasma membrane and then leads to increase of glucose transport through GLUT-4 into the cells [10]. According to these theories, myoinositol could reduce the insulin resistance in pregnancy and myoinositol supplementation could increase the action of endogenous insulin.

The discussed trials indicate that myoinositol supplementation reduces the rate of GDM in both overweight and obese women as well as in women with a family history of GDM [9, 19, 20]. Based on the above research, we can also conclude that myoinositol is well tolerated. This is also confirmed by a study conducted in Italy in which a 29-year-old overweight pregnant woman received 4 g of myoinositol, 3 times per day for 3 weeks. As this case shows, such a high dose of inositol is safe and effective [24]. What is important, in the research presented above, no significant adverse effects of the use of myoinositol have been reported [23].

Some studies have suggested that inositol may play a role in a glycemic control because, in a state of insulin resistance, such as gestational diabetes and polycystic ovary syndrome, there was an increased urinary excretion of inositol metabolites which was positively associated with blood glucose levels. The authors of these studies have indicated that myoinositol supplementation could have an insulin-sensitizing effect [6, 10].

Moreover, the systematic reviews and meta-analyses also underline the significant role of myoinositol in preventing GDM and improving insulin resistance; therefore, myoinositol can reduce the incidence of gestational diabetes [15, 25]. Despite these advantages, there are currently no recommendations for the use of myoinositol among reproductive age women for the prevention of GDM. Although the randomized trials investigating the effect of myoinositol supplementation on the risk of gestational diabetes mellitus gave the encouraging effects in improving insulin resistance, multicenter studies on larger populations, comparing myoinositol with placebo, diet and exercise, and pharmacological interferences, and in double-blind randomized controlled trials are needed to determine the routine use of myoinositol. In addition, these studies should examine the optimal dose, frequency, and timing of supplementation and reveal adverse effects. Such research must be strong enough to assess the potential reduction in the risk of GDM and define if myoinositol supplementation has a long-term effect on maternal, neonatal, and childhood outcomes associated with gestational diabetes mellitus.

## Figures and Tables

**Table 1:** Randomized trials of myoinositol supplementation to prevent GDM.

Study	Type of the study	Intervention	Population	Inclusion criteria	Outcomes
Corrado et al., 2011 [8]	Randomized controlled open-label study	2 g myoinositol +200 mcg folic acid vs. 200 mcg folic acid (placebo) twice/day	MI group: 24 Control group: 45	GDM (OGTT between 24 and 28 weeks)	HOMA-IR decreased significantly greater in MI group (*p* = 0.0001) Adiponectin levels increased in the MI group and decreased in the placebo group (*p* = 0.009)

D'Anna et al., 2013 [9]	Randomized controlled open-label study	2 g myoinositol +200 mcg folic acid vs. 200 mcg folic acid (placebo) twice/day	MI group: 110 Control group: 110	First-degree relatives affected by type 2 diabetes Single pregnancy Prepregnancy BMI < 30 kg/m^2^ Fasting plasma glucose < 126 mg/dL and random glycemia < 200 mg/dL	The incidence of GDM reduced in MI group compared with the placebo group (*p* = 0.04) Mean fetal weight at delivery reduced in MI group.

D'Anna et al., 2015 [19]	Randomized controlled open-label study	2 g myoinositol +200 mcg folic acid vs. 200 mcg folic acid (placebo) twice/day	MI group: 110 Control group: 110	Prepregnancy BMI ≥ 30 kg/m^2^ Age 18-45 Fasting glucose < 126 mg/dL	The incidence of GDM reduced in the MI group compared with the placebo group (*p* = 0.001)

Matarrelli et al., 2013 [17]	Randomized controlled double-blind study	2 g myoinositol +200 mcg folic acid vs. 200 mcg folic acid (placebo) twice/day	MI group: 36 Control group: 39	Single pregnancy Elevated fasting glucose (glycemia ≥ 92 mg/dL and 126 mg/dL) in the first or early second trimester	The incidence of GDM reduced in MI group compared with placebo group (*p* = 0.001) Less necessary of insulin therapy at a later gestational age, higher gestational age at delivery in the MI group

Malvasi et al., 2014 [18]	Randomized controlled double-blind study	2 g myoinositol +400 mcg D-chiroinositol +400 mcg folic acid +10 mg manganese vs. placebo daily	Study group: 24 Control group: 24	Healthy pregnancies 13-24 weeks BMI > 25 kg/m^2^ and <30 kg/m^2^ Age 30-40	Reduction of triglycerides, total cholesterol, LDL-, and HDL-cholesterol

Santamaria et al., 2016 [20]	Randomized controlled open-label study	2 g myoinositol +200 mcg folic acid vs. 200 mcg folic acid (placebo) twice/day	MI group: 110 Control group: 110	Single pregnancy Prepregnancy BMI > 25 kg/m^2^ and <30 kg/m^2^ First trimester fasting glucose ≤ 126 mg/dL and/or random glycemia < 200 mg/dL	The incidence of GDM reduced in the MI group compared with the placebo group (*p* = 0.004)
